# Malaria Among Members of the U.S. Armed Forces, 2025

**Published:** 2026-05-20

## Abstract

Malaria infection remains a potential health threat to U.S. service members located in or near endemic areas due to duty assignments, participation in contingency operations, or personal travel. This report summarizes findings from surveillance of malaria infections among U.S. service members in 2025 and analyzes trends for a 10-year period, from 2016 through 2025. In 2025, 36 cases of malaria were diagnosed among U.S. service members, representing a 12.5% increase from 32 cases reported in 2024. The majority of malaria cases occurred in service members who were male (94.4%), in the active component (80.6%), and serving in the Army (63.9%). Africa was the leading region of acquisition (n=14), primarily for *P. falciparum* infections. A significant 2025 finding is a shift in the predominant species to *P. vivax* , which accounted for 41.7% (n=15) of all cases, a notable increase from 10% of all cases in 2024. Most *P. vivax* cases (80.0%) were acquired in Korea. Seasonality of infection remained consistent, with 72.1% of cases diagnosed May through October. These findings underscore the critical need for continuous surveillance, strict command emphasis on personal protective measures, and region-specific prevention strategies to protect the health of the force and maintain military readiness.

**What are the new findings?**

Findings from 2025 indicate a 12.5% increase in malaria cases among U.S. service members compared to 2024, with a notable increase of new *P. vivax* cases. This change was driven almost entirely by *P. vivax* infections acquired in Korea, while Africa remained the principal source for *P. falciparum* cases.

**What is the impact on readiness and force health protection?**

The rise of *P. vivax* from Korea along with persistent *P. falciparum* infection from Africa not only directly affect force health protection but demonstrate a dynamic, regionally specific threat that requires specialized prevention strategies to maintain military readiness.

Malaria has long posed a significant risk to U.S. military service members and operations. ^1^ Before World War II, the disease was endemic across the southern U.S., prompting the 1942 establishment of the Office of Malaria Control in War Areas—an organization that would later become the Centers for Disease Control and Prevention—to mitigate vector-borne diseases around military installations. ^2^ While this campaign was successful in the elimination of malaria as a public health threat to the U.S. by 1949, the disease remains a persistent risk to the operational readiness of U.S. service members when deployed to endemic tropical and subtropical regions. ^3 - 5^

The risk to military personnel is heightened by operational realities, the emergence of drug-resistant parasites, and inconsistent adherence to preventive measures such as chemoprophylaxis and personal protective equipment. ^6 - 9^ Travel to malaria-endemic regions, especially for foreign-born personnel visiting their countries of origin, also presents a significant medical concern. ^10 - 11^ Studies have shown a significantly higher incidence of malaria in service members and their families with connections to malaria-endemic countries, particularly those from sub-Saharan Africa. This increased risk persists despite universal health coverage and access to pre-travel medical care for this specific population. ^11^

Most human malaria cases are caused by 4 *Plasmodium* species— *P. falciparum, P. vivax, P. malariae* , and *P. ovale* —with *P. falciparum* and *P. vivax* the most significant. *P. falciparum* , found predominantly in Africa, is the most dangerous species, accounting for over 90% of malaria-related deaths, ^12^ while *P. vivax* has the widest geographic distribution, with a high prevalence in Southeast Asia, the Western Pacific, and the Americas. ^13^ These 2 species, *P. falciparum* and *P. vivax* , have distinct epidemiological profiles. A critical difference is the ability of *P. vivax* to cause relapses weeks or even months after initial infection, ^14^ which occurs because the parasite can remain in the liver as hypnozoites, allowing dormant endemicity during the colder, mosquitofree seasons and extending its geographic range into temperate zones, such as the Korean peninsula. ^15^

*MSMR* has published regular updates on malaria's impact on service members since 1999. Current surveillance efforts include the differentiation of malaria types, such as the more lethal *P. falciparum* and relapsing *P. vivax* . This sustained surveillance provides critical data to inform force health protection strategy, and this update continues that mission by describing malaria's epidemiological patterns among U.S. Armed Forces from 2016 through 2025.

## Methods

The surveillance population for this report includes service members of the U.S. Army, Navy, Air Force, Marine Corps, Space Force, and Coast Guard. The surveillance period was January 1, 2016 through December 31, 2025. Records from the Defense Medical Surveillance System (DMSS) were searched to identify qualifying evidence of a malaria diagnosis from reportable medical events (RMEs), hospitalizations, outpatient encounters (in military and non-military facilities), and laboratory results from military facilities.

Case definition criteria for malaria included either 1) an RME record of confirmed malaria, 2) a hospitalization record with a primary diagnosis of malaria, 3) a hospitalization record with a non-primary diagnosis of malaria due to a specific *Plasmodium* species, 4) a hospitalization record with a non-primary diagnosis of malaria plus a diagnosis of anemia, thrombocytopenia, and related conditions, or malariacomplicating pregnancy in any diagnostic position, 5) a hospitalization record with a non-primary diagnosis of malaria plus diagnoses of signs or symptoms consistent with malaria in each diagnostic position preceding malaria, or 6) a positive malaria antigen test plus an outpatient record with a diagnosis of malaria in any diagnostic position within 30 days of the specimen collection date. ^16^ The relevant International Classification of Diseases, 9th and 10th revisions (ICD-9/ICD-10) codes used to identify cases are shown in **Table 1** .

**TABLE 1.** ICD-9 and ICD-10 Diagnosis Codes Used to Define Malaria Cases from Inpatient, Hospitalization Records

		ICD-9	ICD-10
Malaria *Plasmodium* species		
*P. falciparum*	84.0	B50
*P. vivax*	84.1	B51
*P. malariae*	84.2	B52
*P. ovale*	84.3	B53.0
Unspecified	84.4, 84.5, 84.6, 84.8, 84.9	B53.1, B53.8, B54
Anemia	280–285	D50–D53, D55–D64
Thrombocytopenia	287	D69
Malaria complicating pregnancy	647.4	O98.6
Signs, symptoms, or other abnormalities consistent with malaria	276.2, 518.82, 584.9, 723.1, 724.2, 780.0, 780.01, 780.02, 780.03, 780.09, 780.1, 780.3, 780.31, 780.32, 780.33, 780.39, 780.6, 780.60, 780.61, 780.64, 780.65, 780.7, 780.71, 780.72, 780.79, 780.97, 782.4, 784.0, 786.05, 786.09, 786.2, 786.52, 786.59, 787.0, 787.01, 787.02, 787.03, 787.04, 789.2, 790.4	E87.2, J80, M54.2, M54.5, N17.9, R05, R06.0, R06.89, R07.1, R07.81, R07.82, R07.89, R11 * , R16.1, R17, R40 * , R41.0, R41.82, R44 * , R50 * , R51, G44.1, R53 * , R56 * , R68.0, R68.83, R74.0

Abbreviations: ICD-9, International Classification of Diseases, 9th Revision; ICD-10, International Classification of Diseases, 10th Revision; *P., plasmodium* .

* Indicates that any subsequent digit or character is included.

This analysis restricted each service member to 1 episode of malaria per 365-day period. When multiple records documented a single episode, the date of the earliest record was considered the date of clinical onset. Records within 30 days of the clinical onset date were reviewed for evidence of a *Plasmodium* species.

Presumed locations of malaria acquisition were estimated with a hierarchical algorithm: 1) cases diagnosed in a malariaendemic country were considered acquired in that country, 2) RMEs that listed exposures to malaria-endemic locations were considered acquired in those locations, 3) RMEs not listing exposures to malaria-endemic locations but reported from installations in malaria-endemic locations were considered acquired in those locations, 4) cases diagnosed among service members during or within 30 days of deployment or assignment to a malaria-endemic country were considered acquired in that country, and 5) cases diagnosed among service members deployed or assigned to a malaria-endemic country within 2 years before diagnosis were considered acquired in those countries. All remaining cases were considered to have acquired malaria in unknown locations.

## Results

**FIGURE 1.**
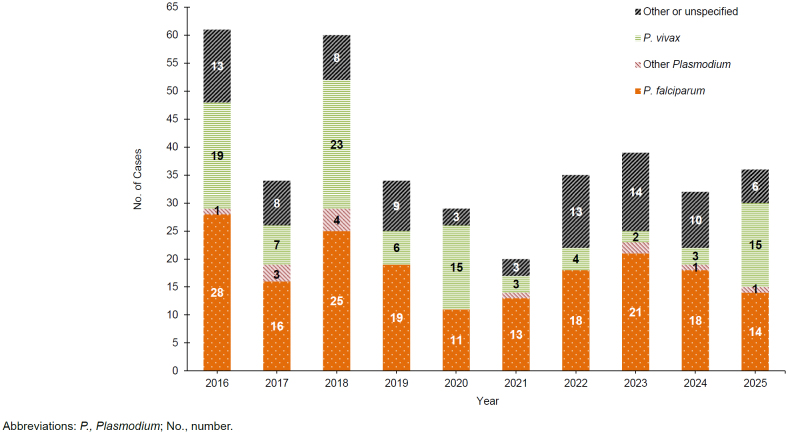
Numbers of Malaria Cases by Species and Calendar Year of Diagnosis or Report, Active and Reserve Components, U.S. Armed Forces, 2016–2025

In 2025, a total of 36 U.S. service members were diagnosed with, or reported to have, malaria **(Table 2)** . The annual total for 2025 represents a 12.5% increase in malaria cases from the 32 cases reported in 2024 **(Figure 1)** . Twenty-eight (77.8%) of the 36 cases in 2025 were identified from RME records. The remaining 8 cases were identified through additional case definition criteria: 5 cases from hospitalization records with a defining diagnosis of malaria in the primary diagnostic position, 2 cases from hospitalization records with a case defining diagnosis of malaria in a non-primary diagnostic position due to a specific *Plasmodium* species or additional diagnoses for malaria-related conditions, and 1 case from a U.S. Department of War laboratory report of a positive malaria antigen test plus 1 outpatient medical encounter for a case-defining diagnosis of malaria in any diagnostic position within 30 days of the specimen collection date (data not shown).

**TABLE 2.** Malaria Cases by *Plasmodium* Species and Selected Demographic Characteristics, U.S. Armed Forces, 2025

	*P. vivax*	*P. falciparum*	Other or Unspecified	Total	
	No.	No.	No.	No.	%
Total	15	14	7	36	100
Component					
Active	13	11	5	29	80.6
Guard, Reserve	2	3	2	7	19.4
Branch of service					
Army	12	7	4	23	63.9
Navy	1	4	0	5	13.9
Air Force	1	1	3	5	13.9
Marine Corps	1	2	0	3	8.3
Sex					
Male	14	13	7	34	94.4
Female	1	1	0	2	5.6
Age, *y*					
<20	2	0	1	3	8.3
20–24	7	2	2	11	30.6
25–29	3	3	2	8	22.2
30–34	2	5	0	7	19.4
35–39	1	1	1	3	8.3
40–44	0	1	1	2	5.6
45–49	0	1	0	1	2.8
50+	0	1	0	1	2.8
Race and ethnicity					
White, non-Hispanic	7	1	3	11	30.6
Black, non-Hispanic	0	13	3	16	44.4
Other	8	0	1	9	25.0

Abbreviations: *P., Plasmodium* ; DMSS, Defense Medical Surveillance System; *y* , years.

As in previous years, the majority of U.S. military members diagnosed with malaria in 2025 were men (94.4%), members of the active component (80.6%), and in the Army (63.9%). No cases were reported in the Space Force or Coast Guard. Non-Hispanic Black service members and individuals ages 20-24 years accounted for the most cases of malaria (44.4% and 30.6%, respectively) **(Table 2)** . Examination of the 28 malaria case records reported as RMEs in 2025 revealed that 5 of the case exposures were classified as deployment-related, 5 as duty-related (but not deployment-related), 10 were non-deployment and non-duty related, while 8 cases were missing exposure classification. Six of the 10 cases classified as non-deployment and non-duty related were documented as acquired in Africa (data not shown).

**FIGURE 2.**
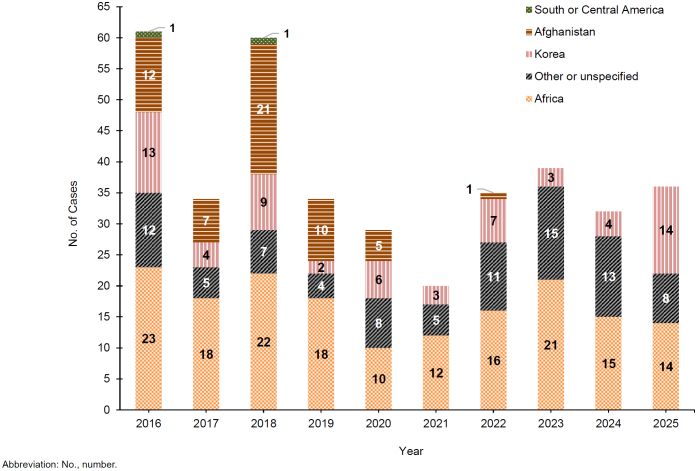
Numbers of Malaria Cases by Location of Acquisition, Active and Reserve Components, U.S. Armed Forces, 2016–2025

During the 2016-2025 surveillance period, malaria cases acquired in Africa (n=169, 44.5%) and other or unspecified locations (n=88, 23.2%) accounted for the largest numbers, followed by Korea (n=65, 17.1%), Afghanistan (n=56, 14.7%), and South and Central America (n=2, 0.5%) **(Figure 2)** . Africa consistently reported the highest numbers of malaria cases throughout the period. Cases in Afghanistan peaked to 21 in 2018, thereafter declining to 0 cases during last 3 years of the surveillance period. Malaria cases were diagnosed or reported in 2025 from 21 different medical facilities: 14 facilities in the U.S., 3 facilities in the Republic of (South) Korea, and 1 facility each in Germany, Africa, and Japan, as well as 1 TRICARE Prime remote location **(Table 3)** .

**TABLE 3.** Number of Malaria Cases by Geographic Location of Diagnosis or Report and Presumed Location of Acquisition, Active and Reserve Components, U.S. Armed Forces, 2025

Location Where Diagnosed or Reported	Korea	Africa	South or Central America	Other or Unknown Location	Total	
	No.	No.	No.	No.	No.	%
AHC Camp Casey, South Korea	9	0	0	0	9	25.0
ACH Camp Humphreys, South Korea	2	0	0	0	2	5.6
Irwin ACH, Fort Riley, KS	0	2	0	0	2	5.6
Madigan AMC, Joint Base Lewis-McChord, WA	2	0	0	0	2	5.6
NMC Portsmouth, VA	0	1	0	1	2	5.6
Travis AFB 60th Medical Group, CA	0	0	0	1	1	2.8
NMC San Diego, CA	0	0	0	1	1	2.8
Evans Carson ACH, Fort Carson, CO	0	1	0	0	1	2.8
NH Jacksonville, FL	0	1	0	0	1	2.8
MacDill AFB 6th Medical Group, FL	0	1	0	0	1	2.8
Robins AFB 78th Medical Group, GA	0	1	0	0	1	2.8
Walter Reed National Military Medical Center, MD	0	1	0	0	1	2.8
Womack AMC, Fort Bragg, NC	0	1	0	0	1	2.8
Alexander T. Augusta Military Medical Center, Fort Belvoir, VA	0	1	0	0	1	2.8
Fairchild AFB 92nd Medical Group, WA	0	1	0	0	1	2.8
Barquist AHC, MD	0	1	0	0	1	2.8
Landstuhl Regional Medical Center, Germany	0	0	0	1	1	2.8
Brian D. Allgood ACH, South Korea	1	0	0	0	1	2.8
NH Okinawa, Japan	0	0	0	1	1	2.8
Expeditionary Medical Facility, Djibouti	0	1	0	0	1	2.8
Pacific Tricare Prime Remote Facility	0	1	0	0	1	2.8
Location not reported	0	0	0	3	3	8.3
Total	14	14	0	8	36	100

Abbreviations: No., number; AMC, Army Medical Center; ACH, Army Community Hospital; AHC, Army Health Clinic; AMC, Army Medical Center; AFB, Air Force Base; NH, Naval Hospital; NMC, Naval Medical Center.

**FIGURE 3.**
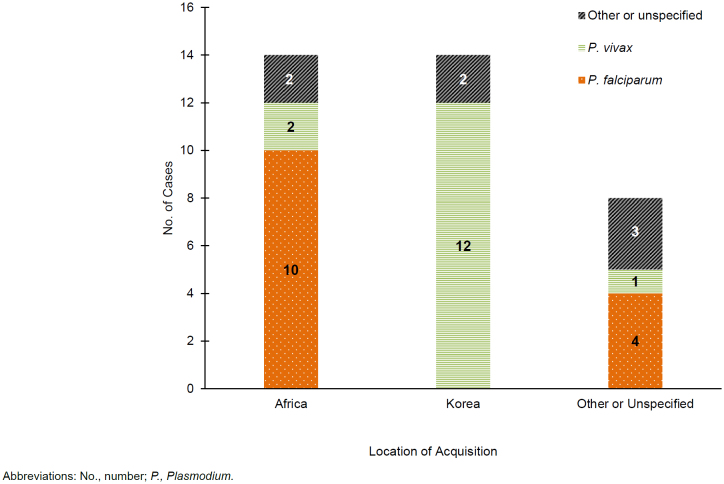
Numbers of Malaria Cases by Species Type and Location of Acquisition, Active and Reserve Components, U.S. Armed Forces, 2025

Most U.S. service member malaria cases in 2025 were caused by *P. vivax* (n=15, 41.7%). Twelve of those 15 *P. vivax* cases were acquired in Korea. The remaining cases were attributed to *P. falciparum* (n=14, 38.9%) and other or unspecified types of malaria (n=7, 19.4%). Most cases acquired in Africa (n=14) were caused by *P. falciparum* (n=10, 71.4%) **(Figure 3)** . The 14 malaria cases acquired in Africa were associated with several countries, including Djibouti (n=3), Ghana (n=3), Cameroon (n=2), Nigeria (n=2), Sierra Leone (n=1), Guinea (n=1), and Tanzania (n=1); 1 case was associated with an unknown African location (data not shown).

**FIGURE 4.**
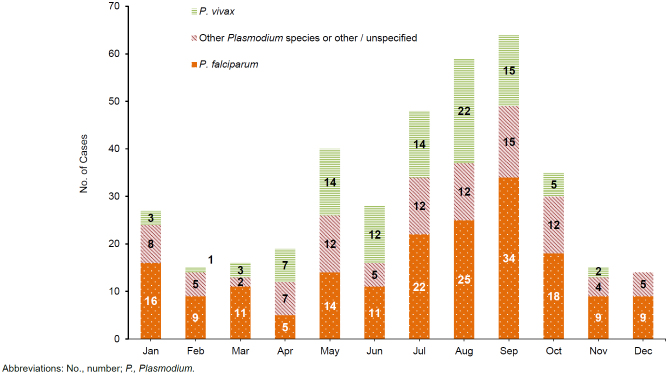
Cumulative Numbers of Malaria Cases by Species Type and Month of Clinical Presentation or Diagnosis, Active and Reserve Components, U.S. Armed Forces, 2016–2025

From 2016 to 2025, malaria caused by *P. falciparum* accounted for the greatest number of cases (183, 48.2%) followed by other or unspecified species (n=99, 26.1%), and *P. vivax* (n=98, 25.8%). Over the 10-year surveillance period, most malaria cases (n=274 / 380, 72.1%) were diagnosed or reported during the 6 months from the Northern Hemisphere middle of spring through the middle of autumn (i.e., May–October) **(Figure 4)** . The proportions of malaria cases diagnosed or reported May–October varied by region of acquisition: Afghanistan (n=48 / 56, 85.7%,), Korea (n=58 / 65, 89.2%), Africa (n=117 / 169, 69.2%), and South and Central America (n=1 / 2, 50.0%) (data not shown).

## Discussion

The number of malaria cases among U.S. service members saw a modest increase in 2025, rising by 12.5% from the previous year. While the total number of cases remains relatively low, these malaria data reveal several key trends and a notable shift in the dominant parasite species, underscoring the persistent threat of malaria to military personnel operating globally. Consistent with historical trends, the demographic profile of malaria cases in 2025 comprised predominantly young, male soldiers from the active component. ^3 - 5^ The majority (77.8%) of cases were identified through routine RMEs, with the remainder captured through hospitalization and laboratory records, highlighting the importance of a multi-faceted surveillance strategy to ensure comprehensive case identification.

Geographically, Africa continues to be a primary region of acquisition for malaria infections, a consistent trend over the last decade. ^3^ The majority of cases acquired in Africa were caused by *P. falciparum* , the most severe form of the parasite. Cases acquired in Africa were traced to at least 7 different countries, reflecting the wide-spread risk across the continent.

Perhaps the most significant finding from the 2025 surveillance data is the dramatic shift in the causative species. Nearly half of all cases were attributed to *P. vivax* , a stark contrast to 2024, when *P. falciparum* accounted for over half of cases — and *P. vivax* only 10%. This change is almost entirely driven by cases acquired in Korea, which accounted for 80% of *P. vivax* infections in 2025. This finding emphasizes the geographically distinct epidemiology of malaria and the specific risks associated with different operational theaters. This risk has long been documented since the Korean War, however, among U.S. and Korean Forces near the Demilitarized Zone. ^17^

The seasonal pattern of malaria diagnoses remains consistent with previous findings, providing predictable opportunity for targeted force health protection measures. Over the 10-year surveillance period, a significant majority (72.1%) of cases were diagnosed May–October, coinciding with the warmer, wetter months that favor mosquito vector activity. ^18^ This trend was particularly pronounced for cases acquired in Korea (89.2%), aligning with the established transmission season for *P. vivax* in temperate zones and reinforcing the need for heightened awareness and preventative measures during these months for personnel in those regions. Vector surveillance programs have shown a correlation between the number of *Anopheles* species positive for *P. vivax* sporozoites with the number of malaria cases and exposure of soldiers from the Republic of Korea soldiers from May through October. ^19^ Even in Africa, where transmission can occur throughout the year, ^18 , 20^ nearly 70% of cases were reported during this same period, underscoring its importance as a peak transmission season globally.

Limitations to this report should be considered when interpreting these findings. Malaria case reporting, especially for reserve components and non-deployment exposures, is likely incomplete, contributing to under-estimation of rates; some cases treated in deployed or non-U.S. military medical facilities may not have been reported or otherwise ascertained at the time of analysis. Malaria diagnoses documented only in outpatient settings without confirmatory testing and not reported as RMEs were not included in this report. Geographic location of malaria acquisition was estimated from reported information, with some cases reporting exposures in multiple malaria-endemic areas and others with no relevant exposure information. Personal travel or deployment to malariaendemic countries was not documented unless specified in RMEs. Limited information on species types in RME records emphasizes the need for more complete attention to documentation of reportable conditions.

These findings emphasize the need for continuous surveillance, regionally specific prevention strategies, and robust diagnostic capabilities to protect U.S. service members from this persistent infectious disease. While the overall burden of malaria within the U.S. military is not significant, 2025 data illustrate a dynamic and evolving threat, with the continued prevalence of *P. falciparum* in Africa posing a significant risk for severe disease and the sharp increase in *P. vivax* from Korea demonstrating a different, but equally important, regional challenge.
